# Deficiency of AMPKα1 Exacerbates Intestinal Injury and Remote Acute Lung Injury in Mesenteric Ischemia and Reperfusion in Mice

**DOI:** 10.3390/ijms22189911

**Published:** 2021-09-14

**Authors:** Hannah V. Hayes, Vivian Wolfe, Michael O’Connor, Nick C. Levinsky, Giovanna Piraino, Basilia Zingarelli

**Affiliations:** 1 Department of Surgery, University of Cincinnati College of Medicine, Cincinnati, OH 45267, USA; LEWISHV@ucmail.uc.edu (H.V.H.); LEVINSNC@ucmail.uc.edu (N.C.L.); 2 Division of Critical Care Medicine, Cincinnati Children’s Hospital Medical Center, and Department of Pediatrics, University of Cincinnati College of Medicine, Cincinnati, OH 45229, USA; vivian.xue@cchmc.org (V.W.); michael.oconnor@cchmc.org (M.O.); giovanna.piraino@cchmc.org (G.P.)

**Keywords:** E-cadherin, occludin, remote acute lung injury, superior mesenteric artery occlusion, syndecan-1

## Abstract

Mesenteric ischemia and reperfusion (I/R) injury can ensue from a variety of vascular diseases and represents a major cause of morbidity and mortality in intensive care units. It causes an inflammatory response associated with local gut dysfunction and remote organ injury. Adenosine monophosphate-activated protein kinase (AMPK) is a crucial regulator of metabolic homeostasis. The catalytic α1 subunit is highly expressed in the intestine and vascular system. In loss-of-function studies, we investigated the biological role of AMPKα1 in affecting the gastrointestinal barrier function. Male knock-out (KO) mice with a systemic deficiency of AMPKα1 and wild-type (WT) mice were subjected to a 30 min occlusion of the superior mesenteric artery. Four hours after reperfusion, AMPKα1 KO mice exhibited exaggerated histological gut injury and impairment of intestinal permeability associated with marked tissue lipid peroxidation and a lower apical expression of the junction proteins occludin and E-cadherin when compared to WT mice. Lung injury with neutrophil sequestration was higher in AMPKα1 KO mice than WT mice and paralleled with higher plasma levels of syndecan-1, a biomarker of endothelial injury. Thus, the data demonstrate that AMPKα1 is an important requisite for epithelial and endothelial integrity and has a protective role in remote organ injury after acute ischemic events.

## 1. Introduction

Mesenteric ischemia is an extremely morbid condition, with mortality in the range of 60–80%, despite advances in diagnosis time and perioperative care. Mesenteric ischemia can be caused by several circumstances, most commonly either an embolic event, thrombotic event or a low-flow state of the intestinal vasculature due to systemic hypotension, major cardiovascular surgery or trauma [1,2]. It has the potential to cause widespread ischemia of the bowel, and, if the insult is not corrected, can progress to necrosis. Although it is imperative to restore blood supply, reperfusion may also represent a secondary injurious hit to the tissue through neutrophil sequestration, oxidative stress and local inflammation [3]. As the condition progresses during ischemia and reperfusion (I/R) injury, the loss of the mucosal barrier function and the systemic inflammatory response can lead to distant organ damage resulting in multiple organ dysfunction syndrome (MODS) [2,3]. Of these distant organs, the lung appears to be the earliest remote organ affected by this pathogenic process [4].

The gastrointestinal epithelial integrity is controlled by the junction complex comprised of the tight junctions (TJ), such as occludins, and the adherent junctions (AJ), such as cadherins [5]. I/R injury perturbs epithelial function also by disrupting these intercellular junctions [6,7,8]. Although this dysfunction can ultimately be ascribed to rapid energy failure and severe alterations in ATP and oxidative stress by free radicals [6,7,8], the precise mechanisms involved in the generation of regulation of junction proteins in the ischemic epithelial cells remain poorly understood.

AMP-activated protein kinase (AMPK) is a key regulator of the cellular homeostatic balance, which is activated through phosphorylation during metabolic cellular stress such as a decreased oxygen supply, decreased glucose or increased AMP/ATP ratio [9]. AMPK is a heterotrimeric enzyme with two regulatory subunits β and γ, and a catalytic subunit α. The α subunit exists in two subforms α1 and α2, with the α1 form being strongly expressed in intestinal, vascular and lung tissue, and the α2 expressed in the heart and striated muscle [10,11,12]. Previous research from our laboratory demonstrated that the activation of the AMPK pathway has an important role in favoring metabolic recovery in injured organs and reducing the systemic inflammatory response during global tissue ischemia following severe hemorrhagic shock [13,14,15,16]. On the contrary, we also demonstrated that AMPKα1 dysfunction during hemorrhagic shock is associated with hemodynamic instability and multiple organ injury [13,16]. Recent studies have also provided evidence that AMPK contributes to the regulation of the assembly of junction proteins in epithelial cells; thus, suggesting a beneficial role of AMPK in the intestinal barrier function [17,18].

To further understand the protective role of AMPK during I/R injury, we sought to investigate the role of AMPKα1 on the processes of local damage and remote distant acute lung injury in a condition of regional mesenteric I/R. Since an impaired intestinal barrier function is one of the key pathological factors of serious complications and negative outcomes after gut I/R injury [2,3], we investigated the biological role of AMPKα1 on affecting the epithelial expression of occludin and E-cadherin. In the loss-of-function studies, we demonstrated that AMPKα1 gene deletion in male mice was associated with an increased susceptibility to mesenteric I/R injury. The AMPKα1 gene deletion also enhanced endothelial dysfunction and increased the susceptibility to distant acute lung injury.

## 2. Results

### 2.1. AMPK Is Activated in Small Intestine Following Mesenteric I/R Injury

**Figure 1:**
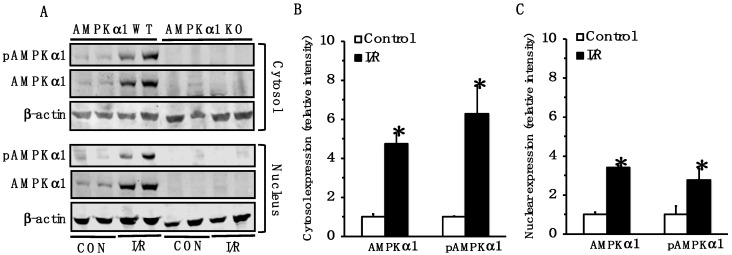
Representative Western blot analysis of pAMPKα1, AMPKα1, and β-actin (used as loading control protein) in small intestine cytosol and nuclear extracts at basal control conditions (CON) and at 4 h after mesenteric ischemia and reperfusion (I/R). (**A**). Quantitative analyses of AMPKα1 and pAMPKα1 expression as determined by densitometry in the cytosol (**B**) and in the nucleus (**C**). Data are mean ± SEM of 3 control mice and 4 mice subjected to I/R injury. Data are expressed as relative intensity units; * *p* < 0.05 versus control mice.

Because of the central role of AMPK in energy homeostasis, we evaluated the intracellular localization and phosphorylation of the catalytic α1 subunit in the small intestine after I/R injury. The cytosol and nuclear content of both total AMPKα1 and its phosphorylated active form, pAMPKα1, increased at 4 h after reperfusion in WT mice when compared to the baseline content of control mice; thus, suggesting the occurrence of AMPK activation under cellular stress conditions. As expected, AMPKα1 was not detected in KO mice (Figure 1).

### 2.2. Genetic Deficiency of AMPKα1 Exacerbates Mesenteric I/R Injury

**Figure 2:**
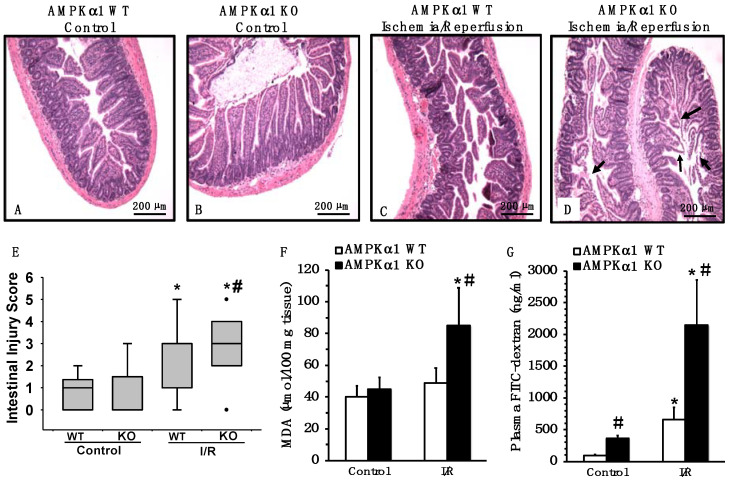
Effect of genetic deficiency of AMPKα1 on gut injury. (**A**–**D**) Representative histology photomicrographs of ileum sections of AMPKα1 WT and KO mice at basal control conditions and at 4 h after mesenteric ischemia and reperfusion (I/R). (**A**) Normal villi and basement membrane in control WT and (**B**) control KO mice. (**C**) Increased edema and hemorrhage and moderate lifting of the epithelial layer from the lamina propria in WT mice subjected to I/R injury. (**D**) Massive disruption to the villi with mucosal ulceration and lifting of the lamina propria (arrows) in KO mice subjected to I/R injury. Magnification ×40. (**E**) Ileum injury score (*n* = 6 mice for each group). Ileum injury was scored from 0 (no damage) to 6 (maximum damage). Box plots represent 25th percentile, median and 75th percentile; error bars define 10th and 90th percentiles; dots define outliers. (**F**) Ileum lipid peroxidation assay by malondialdehyde (MDA). Data represent the mean ± SEM of 10 mice for each group. (**G**) Intestinal permeability measurement by plasma levels of FITC-dextran. Data represent the mean ± SEM of 10 mice for each group; * *p* < 0.05 versus control mice of the same genotype; # *p* < 0.05 versus WT group. Scale bar: 200 μm.

AMPKα1 KO animals had more severe damage in the small intestine after I/R injury when compared to WT mice. The intestinal damage was characterized by a reduction in villus height, villus epithelial lifting/loss and lamina propria swelling (Figure 2A–D). To evaluate the extent of oxidative damage, the lipid peroxidation product malondialdehyde (MDA) was also measured. Consistent with the significant higher pathological score (Figure 2E), AMPKα1 KO animals exhibited higher concentrations of MDA in the small intestine when compared with WT mice (Figure 2F).

### 2.3. Genetic Deficiency of AMPKα1 Is Associated with Increased Epithelial Permeability after Mesenteric I/R

To determine whether AMPKα1 is an important modulator of epithelial barrier integrity, intestinal permeability was measured in WT and KO mice by performing oral gavages with 40 kDa FITC-dextran and measuring the translocation of fluorescence into the plasma. At basal conditions, control AMPKα1 KO mice had higher plasma levels of FITC-dextran compared with control WT mice, indicative of an intestinal leakage already at normal conditions. At 4 h after reperfusion, a significant increase in plasma levels of FITC-dextran was observed in both AMPKα1 WT and KO mice when compared to gene-matched control mice; however, plasma levels of FITC-dextran were five-fold higher in AMPKα1 KO mice than WT mice (Figure 2G).

### 2.4. Genetic Deficiency of AMPKα1 Causes Reduction in Occludin and E-Cadherin Expression after Mesenteric I/R

**Figure 3:**
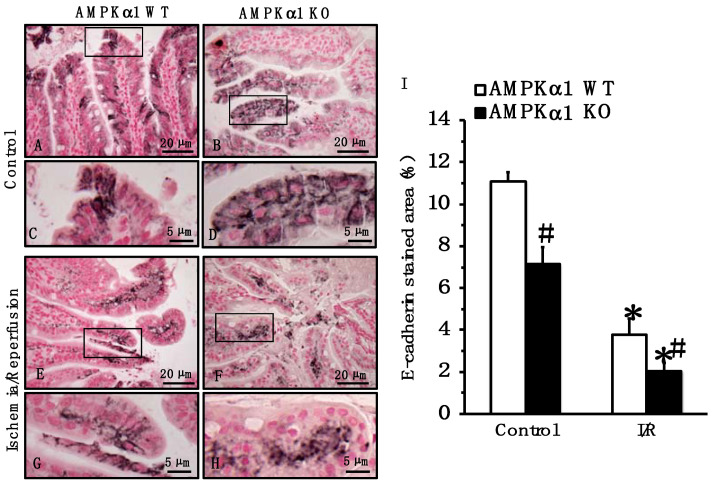
Effect of genetic deficiency of AMPKα1 on gut localization of E-cadherin. (**A**–**H**) Representative photomicrographs of immunostaining of E-cadherin in ileum sections. Black boxes in (**A**,**B**,**E**,**F**) indicate regions of magnified insets shown in (**C**,**D**,**G**,**H**). In control AMPKα1 WT (**A**,**C**) and KO mice (**B**,**D**) E-cadherin was mainly found on the epithelial membrane surface. At 4 h after mesenteric I/R, immunostaining moderately decreased in the villi of WT mice (**E**,**G**) but markedly decreased in KO mice (**F**,**H**). (**I**) Quantitative analysis of stained area. Data are mean ± SEM of 4 animals for each group; * *p* < 0.05 versus control mice of the same genotype; # *p* < 0.05 versus WT group. Scale bars of (**A**,**B**,**E**,**F**): 20 μm. Scale bars of (**C**,**D**,**G**,**H**): 5 μm.

**Figure 4:**
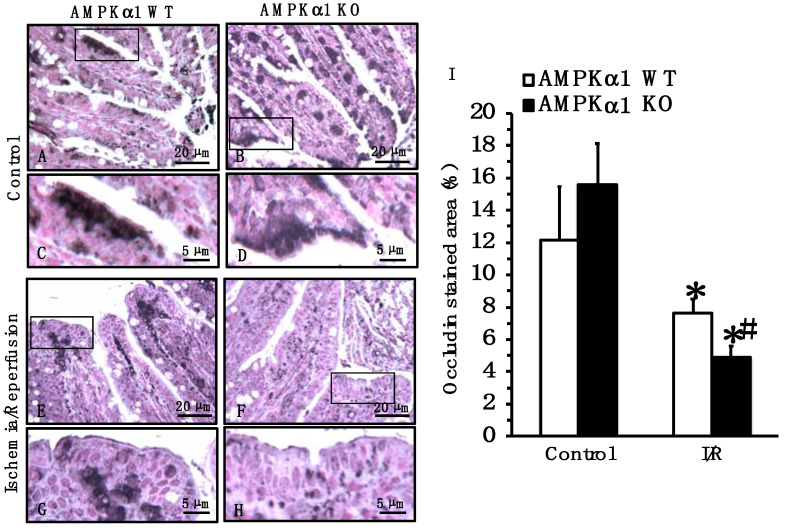
Effect of genetic deficiency of AMPKα1 on gut localization of occludin. (**A**–**H**) Representative photomicrographs of immunostaining of occludin in ileum sections. Black boxes in (**A**,**B**,**E**,**F**) indicate regions of magnified insets shown in (**C**,**D**,**G**,**H**). In control AMPKα1 WT (**A**,**C**) and KO mice (**B**,**D**), occludin was mainly found on the epithelial membrane surface. At 4 h after mesenteric I/R, immunostaining moderately decreased in the villi of WT mice (**E**,**G**) but markedly decreased in KO mice (**F**,**H**) and was mainly confined in the center of the villi. (**I**) Quantitative analysis of stained area. Data are mean ± SEM of 4 animals for each group; * *p* < 0.05 versus control mice of the same genotype; # *p* < 0.05 versus WT group. Scale bars of (**A**,**B**,**E**,**F**): 20 μm. Scale bars of (**C**,**D**,**G**,**H**): 5 μm.

To further investigate the pathophysiological mechanisms of the epithelial barrier dysfunction, we compared the expression and distribution of TJ and AJ proteins. At the immunohistochemical staining of small intestine sections, occludin was localized predominantly at apical surfaces of epithelial cells in small intestine sections of both control AMPKα1 WT and KO mice at basal conditions (Figure 3). E-cadherin was localized in lateral membranes of crypt and apical surfaces of epithelial cells of both control AMPKα1 WT and KO mice at basal conditions; however, stained areas for E-cadherin appeared more pronounced in control AMPKα1 WT when compared to KO mice (Figure 4). A quantitative analysis of immunostaining also confirmed that control AMPKα1 KO mice had a lower expression of E-cadherin, but not occludin, when compared with control WT mice at basal conditions. At 4 h after reperfusion, the expression of occludin and E-cadherin was reduced at the apical and lateral site of the villi, whereas expression was mainly located in the basal portion of epithelial cells in the center of the villi in both AMPKα1 WT and KO mice; however, the loss of the apical component appeared more severe in KO mice when compared to WT littermates (Figure 3 and Figure 4).

### 2.5. Genetic Deficiency of AMPKα1 Exacerbates Lung Injury after Mesenteric I/R

**Figure 5:**
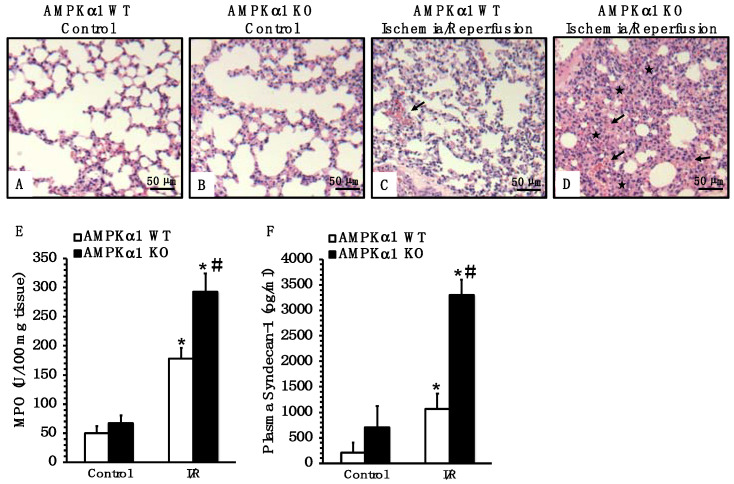
Effect of genetic deficiency of AMPKα1 on lung injury and endothelial damage. (**A**–**D**) Representative histology photomicrographs of lung sections of AMPKα1 WT and KO mice at 4 h after mesenteric ischemia and reperfusion (I/R). (**A**) Normal lung architecture in control WT and (**B**) control KO mice. (**C**) Moderate hemorrhage and inflammatory cell infiltration (arrow) in WT mice subjected to I/R injury. (**D**) Increased alveolar congestion (asterisks), hemorrhage and neutrophil infiltration (arrows) in KO mice subjected to I/R injury. Magnification ×100. A similar pattern was seen in *n* = 6 different tissue sections in each experimental group. (**E**) Lung neutrophil infiltration assay by myeloperoxidase (MPO). Data represent the mean ± SEM of 10 mice for each group. (**F**) Endothelial damage measurement by plasma levels of syndecan-1. Data represent the mean ± SEM of 4–10 mice for each group. * *p* < 0.05 versus control mice of the same genotype; # *p* < 0.05 versus WT group. Scale bar: 50 μm.

Since acute lung injury is the most serious complication of intestinal I/R injury [4], we also evaluated lung histology. At 4 h after reperfusion, AMPKα1 KO mice had more severe lung damage, as characterized by increased alveolar congestion, hemorrhage and neutrophil infiltration compared to WT mice (Figure 5A–D). To further confirm the degree of neutrophil infiltration, we measured the activity of myeloperoxidase (MPO), a lysosomal enzyme specific to neutrophils. Consistent with the histological findings, AMPKα1 KO animals exhibited a higher MPO activity in the lung when compared with WT mice (Figure 5E).

### 2.6. Genetic Deficiency of AMPKα1 Is Associated with Endothelial Glycocalyx Damage after Mesenteric I/R

Since endothelial barrier integrity and capillary leak contribute to distant organ dysfunction during I/R injury [3,17], we measured plasma levels of syndecan-1 as a biomarker of injury to the endothelial glycocalyx [18]. Plasma levels of syndecan-1 increased in both AMPKα1 WT and KO mice at 4 h after reperfusion when compared to baseline levels of control mice; however, KO mice had significant higher levels of syndecan-1 when compared to WT mice, suggesting more severe endothelial barrier damage after mesenteric I/R (Figure 5F).

## 3. Discussion

In the present study, we provided evidence that AMPKα1 is an important requisite for maintaining the intestinal barrier function and mitigating local and distant organ injury caused by mesenteric I/R. Specifically, we showed that an AMPKα1 deficiency in mice had a deleterious impact on intestinal damage and was associated with worse intestinal oxidative stress, an increased paracellular permeability to FITC-dextran and disarrangement of occludin and E-cadherin when compared to WT animals; thus, indicating that AMPK is a crucial regulator of cell junction assembly. Additionally, the deficiency of AMPKα1 was associated with a significant exacerbation of acute lung injury and higher circulating levels of syndecan-1 compared to WT animals; thus, indicating that AMPK may also be important for the preservation of glycocalyx and endothelial barrier.

In several systems, gain- and loss-of-function experiments previously demonstrated the role of AMPK as a critical cytoprotective signal pathway in mitigating I/R injury and oxidative stress by maintaining cellular energy metabolism and promoting cell survival. AMPK activation is indeed an essential component of the adaptive responses to I/R by modulating autophagy and by preventing both necrotic and apoptotic cell death [19]. For example, in myocardial I/R models, the activation of AMPK decreased the reactive oxidative species in cardiac myocytes, whereas mice deficient in the predominantly expressed AMPKα2 subunit had increased cell membrane damage and myocardial necrosis compared to WT mice [20,21]. Additionally, the absence of AMPKα2 resulted in the development of ischemic contractures and delayed recovery in a model of low-flow I/R in perfused hearts [22]. Similarly, preconditioning or pharmacological treatment with AMPK activators in acute kidney I/R reduced reactive oxygen species generation and ameliorated renal vasodilation and tissue metabolism [23,24]. In our current study, we demonstrated for the first time that the expression and activation of AMPKα1 increased both in the cytosol and the nuclear compartments of the small intestine after mesenteric I/R in AMPKα1 WT mice; thus, suggesting a mechanism to counteract metabolic disturbances. This increase was associated with milder injury when compared to AMPKα1 KO mice. In line with these cytoprotective effects of AMPK, we previously showed that the pharmacological activation of AMPK exerted lung and cardiac therapeutic effects and improved the hemodynamic compensatory mechanisms in murine models of global I/R injury by severe hemorrhage [13,14,15,16]. Other cytoprotective mechanisms of AMPK activity were also related to its ability to modulate gene expression of antioxidant defenses. The pharmacological activation of AMPK has been proposed to be beneficial in the endothelium through decrease in oxidative and apoptotic genes in addition to bioenergetic effects [25,26,27]. In previous studies, we also demonstrated that the pharmacological activation of AMPK ameliorated liver injury in mice subjected to sepsis by regulating the gene transcription of apoptotic proteins, mitochondrial structural and transport proteins, metabolic and antioxidant enzymes [28]. In the current study, a remarkable epithelial loss was observed in AMPK KO mice when compared to WT mice; thus, proving the occurrence of cell death in the absence of AMPK. The excessive vulnerability to injury in mice with AMPKα1 ablation was associated with an increased intestinal content of MDA, a product of lipid peroxidation indirectly related to the degree of oxidative stress [29]; thus, suggesting that AMPKα1 is important for regulating antioxidant mechanisms in the intestinal epithelium.

The intestinal barrier dysfunction is a key factor in the aggravation of the deleterious complications of intestinal I/R, as it may lead to bacterial translocation from the gut, secondary organ injuries and MODS, and death. The principal determinant of intestinal permeability and trans-epithelial transport is the TJ complex, which is located at the apex of the lateral plasma membranes between adhering cells [6,7,8]. Occludin is one of the key TJ proteins that contributes to the maintenance of an intact intestinal epithelium. Immediately below the TJ proteins are the cadherin-rich AJ proteins that mediate strong cell-to-cell adhesion and have functional roles in forming and regulating the epithelial barrier through cell signaling and the regulation of gene transcription [30,31]. Intracellular internalization and the subsequent degradation of TJ and AJ proteins have been described as pivotal mechanisms of barrier dysfunction [32,33,34]. Our current study is the first to suggest that the proper activity of AMPKα1 is an essential factor for the intestinal epithelial barrier function through the regulation of integrity and stability of the junction network during I/R injury. We demonstrated that the deletion of AMPKα1 in KO mice augmented intestinal permeability to FITC-dextran both at control conditions and after I/R in comparison to WT mice; thus, indicating that AMPK is a key factor in the maintenance of gut epithelial integrity. When investigating the structural changes at immunostaining, we observed that mesenteric I/R led to the loss of occludin and E-cadherin on the apical epithelial surface. Interestingly, after I/R, both junction proteins appeared centrally located. The surface disappearance was more evident in AMPKα1 KO mice when compared to WT mice.

These findings were consistent with previous in vivo reports showing that the specific deletion of AMPK in epithelial cells augmented intestinal permeability and exaggerated the inflammatory response in experimental colitis [35]. Similarly, in vitro studies in Caco-2 cells demonstrated that AMPK deletion induced a delay in junction proteins re-assembly and re-localization at the plasma membrane during calcium switch, leading to impairment in the paracellular permeability [36]. It is important to note that the phenomenon of internalization has been reported to occur in events of variations of the cellular energy status and to precede protein degradation. In canine kidney cells, the majority of surface-accessible E-cadherin was internalized within the first 60 min of ATP depletion and was, subsequently, subjected to intracellular degradation [37]. How AMPK activation regulates the stability of TJ and AJ proteins remains to be elucidated. It has been proposed that AMPK regulate the assembling of junction proteins at multiple mechanistic levels. It has been shown that AMPK directly phosphorylates proteins which are localized at the plasma membrane and are involved in the regulation of junction proteins, such as protein kinase C, which in turn phosphorylates occludin and regulates its localization at the membrane [38,39]. In addition to occludin, AMPK signaling has been associated with the abundance of other TJ proteins, such as claudins and zonula occludens-1 and with the regulation of paracellular permeability in epithelial cells [40,41]. Previous studies have also shown that AMPK is recruited to the E-cadherin complex in response to a mechanical force in epithelial cells, where it provides ATP to reinforce the adhesion complex [42]. Of note in our study, we also observed that control AMPKα1 KO mice had a lower expression of E-cadherin, but not occludin, when compared to WT mice. This downregulation at basal conditions was consistent with the presence of FITC-dextran leakage in AMPKα1 KO mice. Thus, taken together, these findings suggest that both the constitutive baseline activation as well as stress-induced activation of AMPK contributed to proper transmembrane assembly.

Acute lung injury is one of the most important components of MODS triggered by intestinal I/R injury and represents a leading cause of death in critically ill patients with a high mortality of approximately 40% [4]. Several pathophysiological events during intestinal injury may affect the lung. In addition to hyperpermeability, which can result in bacterial translocation into the systemic circulation, mesenteric I/R is also associated with release in the lymphatic ducts of toxic factors, such as cytokines, lipases and free fatty acids leading to the activation of inflammatory neutrophils in the lung [43]. In our study, the magnitude of lung damage and neutrophil sequestration was higher in AMPKα1 KO mice when compared to WT mice and it was consistent with the disruption of the intestinal epithelial barrier. Since endothelial dysfunction is linked to leukocyte infiltration into surrounding tissues and the loss of vascular integrity and permeability [17], we also evaluated the circulating levels of syndecan-1, a biomarker of endothelial glycocalyx shedding [18]. In our study, AMPKα1 KO mice exhibited a significant increase in circulating levels of syndecan-1 when compared to WT mice. It is important to consider that in clinical studies circulating levels of syndecan-1 showed the strongest associations with neutrophil activation biomarkers and adverse clinical outcomes in patients with respiratory failure [44]. Thus, taken together, our findings support the notion that the metabolic effects of AMPKα1 in different cell types may contribute to regulate inflammation during I/R injury and they may extend to the maintenance of endothelial integrity. The results of the present study also confirmed the role of AMPKα1 in the protection of lung injury as previously demonstrated in models of tissue hypoxemia, such as hemorrhagic shock [13,15,16].

In conclusion, our findings provide new insights into the mechanisms underlying intestinal I/R injury and suggest that the proper activation of AMPKα1 is an important defense mechanism to counteract local and systemic inflammation and to maintain intestinal and endothelial barrier function, and it may be considered as an adjunct therapeutic target.

## 4. Materials and Methods

### 4.1. Murine Model of Mesenteric I/R Injury

The study complied with the *Guide for the Care and Use of Laboratory Animals* published by the U.S. National Institutes of Health (8th Ed., 2011; Bethesda, MD, USA) and met the approval of the Institutional Animal Care and Use Committee of the Cincinnati Children’s Hospital Medical Center. Homozygous AMPKα1 wild-type (*Ampkα1*^+/+^ WT) and AMPKα1 knock-out (*Ampkα1^−/−^* KO) mice were established on a C57BL/6 genetic background by the crossbreeding (>10 generations) of a breeding pair kindly provided by Dr. Benoit Viollet of the University of Paris Descartes, Paris, France [45]. For these experiments, male AMPKα1 WT and KO mice were generated by a breeding scheme utilizing heterozygous mutant mice. All mice were singly housed to avoid fighting and provided with additional enrichment to minimize social stress. Mice were allowed free access to water and a maintenance diet in a 10 h light/14 h dark cycle, with room temperature at 21 ± 2 °C. Mice were used at the age of 3–5 months and assigned to the different experimental groups after routine genotyping by qualitative PCR by using the following primer sequences: forward primer 5′-TTAGACCTGAAGAGATGGCACCC-3′; reverse primer 5′-GACGCAACTGTCAAAGAGCAAATC-3′. Mice were anesthetized by inhalation with 1–3% isoflurane. A midline laparotomy was performed, and the intestines were externalized. The superior mesenteric artery was dissected from the mesentery and was clamped along with its proximal branches using three 60 g pressure microvascular clips. The small bowel and cecum were covered with saline-soaked gauze and ischemic time allowed for 30 min. After 30 min, the clips were removed, the intestines placed back into the abdominal cavity and abdominal incision was closed. To minimize pain at the surgical incision site, mice received 0.25% bupivacaine (1 mg/kg) subcutaneously. Mice were also resuscitated subcutaneously with 35 mL/kg 0.9% sodium chloride solution immediately after the surgical procedure and returned to their cage with access to food and water *ad libitum*. Control mice did not undergo any surgical procedure. Animals were euthanized at 4 h after reperfusion. Blood, lung and small intestine were collected for biochemical assays.

### 4.2. Cytosol and Nuclear Protein Extraction

Distal ileum samples were homogenized in a buffer containing 0.32 M sucrose, 10 mM Tris-HCl (pH 7.4), 1 mM EGTA, 2 mM EDTA, 5 mM NaN3, 10 mM β-mercaptoethanol, 2 µM leupeptin, 0.15 µM pepstatin A, 0.2 mM phenylmethanesulfonyl fluoride, 50 mM NaF, 1 mM sodium orthovanadate and 0.4 nM microcystin. Samples were centrifuged at 1000× *g* for 10 min at 4 °C and the supernatants collected as cytosol extracts. The pellets were then solubilized in Triton buffer (1% Triton X-100, 250 mM NaCl, 50 mM Tris HCl at pH 7.5, 3 mM EGTA, 3 mM EDTA, 1 mM phenylmethanesulfonyl fluoride, 0.1 mM sodium orthovanadate, 10% glycerol, 2 mM p-nitrophenyl phosphate, 0.5% NP-40 and 46 µM aprotinin). The lysates were centrifuged at 15,000× *g* for 30 min at 4° C and the supernatant collected as nuclear extracts.

### 4.3. Western Blot Analysis

Cytosol and nuclear content of AMPKα1 and its phosphorylated form pAMPKα1 were determined by immunoblot analyses. Extracts were heated at 70 °C in equal volumes of 4× Protein Sample Loading Buffer. Forty μg of protein were loaded per lane on a 10% Bis-Tris gel. Proteins were separated electrophoretically and transferred to nitrocellulose membranes. For immunoblotting, membranes were blocked with Odyssey blocking buffer (LI-COR Biotechnology, Lincoln, NE, USA) and incubated with primary antibodies from Santa Cruz Biotechnology, Dallas, TX, USA: rabbit anti-AMPKα1 and rabbit anti-pAMPKα1 (Ser 496) antibodies; β-actin was concomitantly probed with mouse anti-β-actin as a loading control for both cytosol and nuclear proteins. Membranes were washed in PBS with 0.1% TWEEN 20 and incubated with near infrared fluorescent dye-conjugated secondary antibodies (IRDye goat anti-rabbit and anti-mouse, and donkey anti-goat IgG; LI-COR Biotechnology). The Odyssey LI-COR scanner (LI-COR Biotechnology) was used for detection. Fold changes of relative intensity of proteins were calculated versus mean value of control mice upon data normalization with β-actin by NIH ImageJ 1.53k software [46].

### 4.4. Histological Analysis of Intestinal Injury

Distal ileum was flushed with saline solution to clear luminal content and placed immediately in 10% neutral buffered formalin. The tissue was embedded in paraffin, sectioned and stained with hematoxylin and eosin. Sections were evaluated by 4 blinded independent observers by light microscopy. Intestinal injury was graded using a villous injury severity scale [47,48] with a score of 0 equaling normal mucosa; 1 representing vacuolization at the villus tip and development of subepithelial Gruenhagen’s space; 2 indicating moderate lifting of the epithelial layer from the lamina propria and extension of Gruenhagen’s space; 3 demonstrating increased vacuolization from the tip to the middle of the villus and massive subepithelial lifting; 4 corresponding to vacuolization and epithelial lifting from the tip to the lower portion of the villi; 5 representing disintegration of the lamina propria and mucosal ulceration.

### 4.5. In Vivo Measurement of Intestinal Permeability

In a separate group of mice, intestinal mucosal permeability to fluorescein isothiocyanate-conjugated dextran (40 kDa FITC-dextran, MilliporeSigma, Burlington, MA, USA) was measured in vivo. Mice were solid food restricted with free access to water 6 h before surgery. Thirty minutes prior to the induction of anesthesia, animals underwent oral gavage with 20 mL/kg of 40 kDa FITC-dextran (25 mg/mL) in phosphate-buffered saline solution. Animals then underwent the intestinal I/R procedure as described above. At 4 h after reperfusion, blood was collected and centrifuged at 3000 rpm at 4 °C for 20 min. Plasma samples were analyzed by fluorescence spectrophotometry at excitation wavelength of 485 nm and emission wavelength of 525 nm. Concentration of FITC-dextran was determined by creating a standard curve with serial dilution in normal mouse plasma [49].

### 4.6. Malondialdehyde Assay

Malondialdehyde (MDA) activity was measured as an index of lipid peroxidation. Intestinal tissue was homogenized in 1.15% potassium chloride solution. Then, 8.1% sodium dodecyl sulfate, 20% acetic acid, 0.8% thiobarbituric acid and distilled water were added to the homogenates. The mixture was boiled for 1 h at 95 °C and, then, centrifuged at 3000 rpm for 10 min. The absorbance was measured by spectrophotometry at 532 nm and expressed as MDA in µmol per 100 mg tissue [50].

### 4.7. Immunohistochemistry

For immunohistochemical detection of junction proteins, 5 μm thick intestinal sections were deparaffinized, rehydrated and, subsequently, blocked with 2% normal goat serum at room temperature for 2 h. Sections were then incubated with primary antibodies against occludin (1:40; Life Technology/Thermo Fischer Scientific, Whaltham, MA, USA) and against E-cadherin (1:400; Cell Signaling, Danvers, MA, USA) overnight at 4 °C. Specific labelling was detected by incubating sections for 30 min with a biotinylated goat anti-rabbit secondary antibody (Vector Laboratories, Burlingame, CA, USA) and amplified with avidin–biotin peroxidase complex (ABC Vectastain Elite kit, Vector Laboratories) after quenching endogenous peroxidase with 3% H_2_O_2_ in water for 5 min. Immunoreactivity was identified with diaminobenzidine (MilliporeSigma) as a peroxidase substrate, using Nuclear Fast Red (MilliporeSigma) as a counterstain. Samples were imaged using an Olympus BX40 microscope fitted with a digital camera and Q Capture Pro 7 software (QImaging). Quantitative analysis of immunostaining was performed by using NIH ImageJ 1.53k software and percentage of positive areas were calculated by measurement of threshold on 8-bit images [46].

### 4.8. Histological Analysis of Lung Injury

Lungs were fixed in 10% neutral buffered formalin and embedded in paraffin. Sections were stained with hematoxylin and eosin and evaluated by two independent observers. Lung injury was analyzed on the following histologic features: alveolar capillary congestion, infiltration of red blood cells and inflammatory cells into the airspace, alveolar wall thickness and hyaline membrane formation [13].

### 4.9. Measurement of Myeloperoxidase Activity

Myeloperoxidase (MPO) activity, an index of neutrophil infiltration, was measured in the lung. Lung tissue in a solution containing 0.5% hexa-decyl-trimethyl-ammonium bromide dissolved in 10 mM potassium phosphate buffer (pH 7) and centrifuged for 30 min at 4000× *g* at 4 °C. An aliquot of the supernatant was allowed to react for 5 min with a solution of tetra-methyl-benzidine (1.6 mM) and 0.1 mM H_2_O_2_. After 3 min, the reaction was halted using 2 M acetic acid. The rate of change in absorbance was measured by spectrophotometry at 650 nm. MPO activity was defined as the quantity of enzyme degrading 1 μmol of hydrogen peroxide/minute at 37 °C and expressed in units per 100 mg tissue [51].

### 4.10. Plasma Levels of Syndecan-1

Plasma syndecan-1 concentration, an index of endothelial injury, was determined using a mouse syndecan-1 sandwich-type enzyme-linked immunosorbent assay kit (Boster Biological Technology, Pleasanton, CA, USA) according to the manufacturer’s instructions.

### 4.11. Statistical Analysis

Statistical analysis was performed using SigmaPlot 14.0 (Systat Software, San Jose, CA, USA). Data in figures and text are expressed means ± SEM or median with 25th and 75th percentile of *n* observations (*n* = 4–10 animals for each group). The results were examined by analysis of variance followed by the Student–Newman–Keuls correction post hoc *t*-test. Statistical analysis of damage scores was performed using the non-parametric Mann–Whitney test. A value of *p* < 0.05 was considered significant.

## Data Availability

The data presented in this study are available on request from the corresponding author. The data are not publicly available due to privacy.

## Abbreviations

AJ	adherent junction
AMP	adenosine monophosphate
AMPK	AMP-activated protein kinase
ATP	adenosine triphosphate
FITC-dextran	fluorescein isothiocyanate-conjugated dextran
I/R	ischemia and reperfusion
KO	knock-out
MDA	malondialdehyde
MODS	multiple organ dysfunction syndrome
MPO	Myeloperoxidase
PCR	polymerase chain reaction
TJ	tight junction
WT	wild-type
